# Comparison of Quality of Life Between Patients with Advanced Heart Failure and Heart Transplant Recipients

**DOI:** 10.21470/1678-9741-2020-0402

**Published:** 2021

**Authors:** Wágner do Nascimento Carvalho, Gustavo dos Santos Alves Maria, Karla Cordeiro Gonçalves, Anna Letícia Miranda, Maria da Consolação Vieira Moreira

**Affiliations:** 1 Faculdade de Medicina, Universidade Federal de Minas Gerais, Belo Horizonte, Minas Gerais, Brazil.; 2 Hospital das Clínicas, Universidade Federal de Minas Gerais, Belo Horizonte, Minas Gerais, Brazil.; 3 Department of Internal Medicine, Clínica Médica, Faculdade de Medicina, Universidade Federal de Minas Gerais, Belo Horizonte, Minas Gerais, Brazil.

**Keywords:** Heart Transplantation, Heart Failure, Cardiomyopathies, Quality of Life, Health-Related Quality of Life, Surveys and Questionnaires

## Abstract

**Introduction:**

Heart transplantation is the treatment indicated for patients with advanced and refractory heart failure (HF). The transplant is expected to increase survival and improve the level of health-related quality of life (HRQoL). The aim of this study was to compare the level of HRQoL, as well as social and clinical variables, between patients with advanced HF and heart transplant (HT) recipients.

**Methods:**

This is a cross-sectional study, conducted at a Brazilian university hospital, during outpatient consultations. The level of HRQoL was assessed using the World Health Organization Quality of Life-Bref questionnaire. Descriptive statistics were used to analyze the data, and the comparison of the level of HRQoL was performed using the Mann-Whitney U test.

**Results:**

Two hundred sixty-two patients participated in the study. Seventy-nine of them had advanced-stage HF and 183 were HT recipients. Compared to patients with advanced HF, HT recipients had a better level of HRQoL, were less frequently absent from work due to health problems, had higher income, used a higher number of medications, and there was a higher percentage of retirees among them (*P*-value < 0.001).

**Conclusion:**

In every comparison, HT recipients showed a better level of HRQoL than patients with advanced HF, along with less absence from work and higher income. These results suggest that heart transplantation can improve HRQoL and survival of patients with advanced HF.

**Table:** 

Abbreviations, acronyms & symbols	
HF	= Heart failure
HRQoL	= Health-related quality of life
HT	= Heart transplant
MW	= Minimum wage
QoL	= Quality of life
WHOQOL-Bref	= World Health Organization Quality of Life - Bref

## INTRODUCTION

Heart transplantation is the treatment indicated for patients with advanced heart failure (HF) that are refractory to optimized treatment and have poor prognosis. Besides survival improvement, health-related quality of life (HRQoL) is also an objective of heat transplantation ^[1,2]^. HRQoL covers aspects related to the individual’s self-perception of well-being, which can be affected in situations such as coping with a disease ^[3]^. However, complications after transplantation - such as rejections, infections, graft failure, cancer, and allograph vasculopathy - limit survival ^[2,4]^ and, consequently, may affect the patients’ levels of HRQoL.

Quality of life (QoL) is a multidimensional concept, which assesses the individual perception of one’s own position in life, taking into consideration the cultural setting and the system of values in which him or her is inserted ^[5]^. HRQoL is a subdivision of this concept, which “focuses on the impacts of medical interventions from an individual perspective” (Tackmann and Dettmer, 2020) ^[6]^. Although heart transplantation is considered a definitive therapy for HF, it is also related with clinical complications that can affect HRQoL, such as rejections, infections, graft failure, kidney disease, cancer, and allograph vasculopathy ^[7]^; therefore, it is relevant to measure and compare HRQoL in both pre- and post-transplantation settings, to evaluate if it is adequately being improved with the procedure.

The scarcity of organs is one of the most important factors that limit the performance of the heart transplant (HT) ^[1,8]^. Ventricular assist devices, which could be used as a bridge for heart transplantation or as a target therapy for improvement of both survival and HRQoL ^[2,9]^, are not routinely available in many countries.

The HRQoL analysis is an important measurement of results in the post-transplantation evaluation ^[10]^. By comparing HT recipients with advanced HF patients, it is possible to understand how patients evolve their lifestyle after heart transplantation, and if it significantly improves the HRQoL. The objective of this study, which was carried out in a Brazilian university hospital, was to compare the level of HRQoL, as well as social and clinical variables, between patients with advanced HF and HT recipients.

## METHODS

This is a cross-sectional study, approved by the Universidade Federal de Minas Gerais Research Ethics Committee, under the approval number 2.510.460. It was carried out in a Brazilian university hospital from March 2018 to April 2019. Study participants were patients that underwent heart transplantation between 2006 and 2018 and patients with advanced HF (functional classes of New York Heart Association III and IV), candidates or not to heart transplantation, who were followed up at the cardiology outpatient clinic from March 2018 to April 2019. Exclusion criteria were age < 18 years, diagnosis of dementia, and cognitive impairment.

The number of patients with advanced HF seen in the outpatient clinic is dynamic, varying according to factors such as referral by the central regulation of the public health system and number of heart transplantation procedures. The population of HT recipients was initially composed of 198 patients, but only 192 met the inclusion and exclusion criteria. Four patients refused to participate in the study and five patients could not be contacted during the study period. The final sample was composed of 262 patients, divided into two groups: 79 with advanced HF and 183 HT recipients.

The study participants were interviewed in a research room, by one of the four researchers responsible for the interviews. Prior to the beginning of data collection, all of them were specifically trained for the interviewing process and participated in the discussion of the documents used for standardization.

Before the beginning of the interviews, the patients signed a free and informed consent form and were informed by the researcher that they could use the time they needed to answer the questions. The patients could also interrupt the interviewer to clarify themselves when they did not understand the asked question. A clinical form, prepared by the authors, was used as the data collection source. It contained fields with social and clinical data, which were collected during the interview and after it, using information of the patients’ medical records.

The level of HRQoL was assessed using the World Health Organization Quality of Life-Bref (WHOQOL-Bref) questionnaire, created by the World Health Organization, translated and validated for use in Brazil ^[5]^. The WHOQOL-Bref is composed of 26 questions: two of them addresses general questions about the perception of QoL and health, and the 24 remaining questions are classified in four domains (physical health, psychological, social relationships, and environment). Each of the 26 questions is scored from 1 to 5 on a Likert scale. The raw score from each of the 24 facets is converted into a transformed score, that can vary from 0 to 100 points; the higher the score, the better the level of HRQoL ^[5]^. The questionnaire was used exclusively for this study and the interviews were performed from March 2018 to April 2019 in the cardiology outpatient clinic of the institution.

In the statistical analysis, categorical variables were analyzed by absolute and relative frequency. Continuous variables did not show normal distribution by the Shapiro-Wilk test, being therefore presented as median, first, and third quartiles. Continuous variables were compared between the two independent groups using Mann-Whitney U non-parametric test. For categorical variables, Pearson's chi-square test was firstly applied; when its assumptions were not satisfied, Fisher's exact test was used. The comparison of the level of HRQoL between the two independent groups was performed using the Mann-Whitney U non-parametric test. Statistical analyses were performed using a significance level of 0.05. The Statistical Package for the Social Sciences (IBM SPSS Statistics, USA) software, version 18.0, was used for data analysis.

## RESULTS

A total of 262 patients participated in this study. One hundred eighty-three of them were HT recipients and 79 had advanced stage HF, being candidates or not for heart transplantation. Most patients were male (n=167, 63.7%), non-white (n=199, 76%), retired (n=174, 66.4%), and had up to eight years of formal education (n=165, 62.9%). Their median age was 53 (43 - 59) years. Two hundred and seven (79%) patients had a per capita income inferior to two Brazilian minimum wages (one Brazilian minimum wage currently corresponds to approximately US$250 monthly). The main etiology of HF was Chagas cardiomyopathy (n=105, 41%) followed by idiopathic dilated cardiomyopathy (n=71, 27.1%). The categorization of the two groups of patients, according to social and clinical variables, is shown in Table 1.

**Table 1:** Comparison of social and clinical variables between patients with advanced heart failure and heart transplant recipients.

Variable	HF patients	HT recipients	*P*-value
**Sex^a^**			
Male	63 (67.1)	114 (62.3)	0.459
Female	26 (32.9)	69 (67.7)	
Age^b^ (years)	52 (43 - 58)	53 (42 - 60)	0.590
**Ethnicity**			
White	17 (21.5)	46 (25.1)	0.529
Non-white	62 (78.5)	137 (74.9)	
**Marital status^c^**			
Married/stable union	56 (70.9)	127 (69.4)	
Single	9 (11.4)	36 (19.7)	0.199
Divorced	8 (10.1)	13 (7.1)	
Widow/widower	6 (7.6)	7 (3.8)	
**Education level^a^**			
Illiterate	4 (5.1)	6 (3.3)	
Incomplete elementary school (< 8 years)	37 (46.8)	76 (41.5)	
Complete elementary school (8 years)	19 (24.1)	33 (18)	0.273
Complete high school (11 years)	12 (15.2)	49 (26.8)	
University education	7 (8.9)	19 (10.4)	
**Occupation^c^**			
Retired	40 (50.6)	134 (73.2)	
Away from work due to health problems	26 (32.9)	16 (8.7)	
Household work	6 (7.6)	8 (4.4)	< 0.001
Student	-	6 (3.3)	
Others	7 (8.9)	19 (10.4)	
**Per capita income^a^**			
< 1 MW*	51 (64.6)	71 (38.8)	
Between 1 and 2 MW	18 (22.8)	67 (36.6)	< 0.001
> 2 MW	10 (12.7)	45 (24.6)	
**Etiology of heart failure^a^**			
Chagas cardiomyopathy	26 (32.9)	79 (43.2)	
Idiopathic dilated cardiomyopathy	21 (26.6)	50 (27.3)	0.315
Ischemic cardiomyopathy	17 (21.5)	30 (16.4)	
Others	15 (19)	24 (13.1)	
Number of current medications^b^	6 (5 - 8)	8 (7 - 10)	< 0.001

Frequency (%); Median (1^st^ quartile - 3^rd^ quartile);

a Pearson's Chi-Square test;

b Mann-Whitney U test

c Fisher's exact test;

* one MW currently corresponds to approximately US$250 monthly HF=heart failure; HT=heart transplant; MW=minimum wage

The occupational status of HT recipients was significantly different from the one of HF patients (*P*-value<0.001): there was a higher percentage of retirees among HT recipients, a lower percentage of absence from work due to health problems, and they had more years of education. HT recipients also had a higher per capita income (*P*-value 0.001), with almost the double of percentage of patients receiving ≥ 2 minimum wages. These results are shown in Table 1.

Pharmacological therapy also differed between the two groups of patients (*P*-value<0.001). Although patients with advanced HF are known to use several drugs, HT recipients used an even higher number of medications, due to the need to add immunosuppressive agents.

**Fig. 1:**
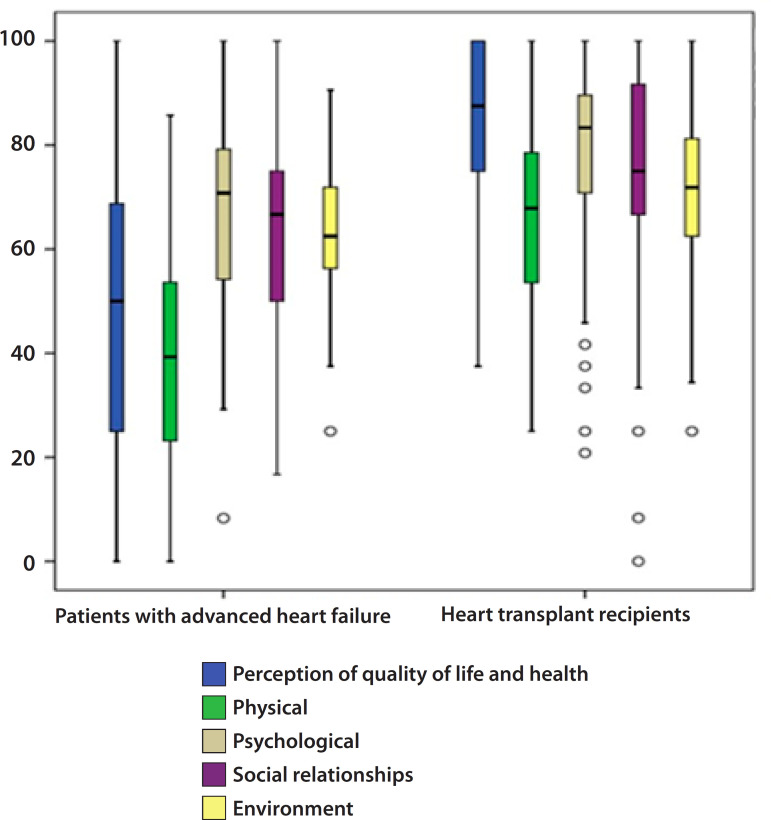
Perception of the level of quality of life and health - physical, psychological, social relationships, and environment domains - in the World Health Organization Quality of Life-Bref questionnaire of patients with advanced heart failure and heart transplant recipients.

The median score of perception of QoL and health was higher in the HT recipients group (*P*-value<0.001), as well as every domain of WHOQOL-Bref, as shown in Figure 1 and Table 2.

**Table 2:** Comparison between self-perception of the level of quality of life and health (according to the World Health Organization Quality of Life-Bref questionnaire) between patients with advanced heart failure and heart transplant recipients.

Variable	HF patients	HT recipients	*P*-value
**Perception of the level of quality of life and health**	50 (25 - 68.7)	87.5 (75 - 100)	< 0.001
**Domains**			
Physical health	39.3 (23.2 - 53.6)	67.8 (53.5 - 78.5)	< 0.001
Psychological	70.8 (54.2 - 79.2)	83.3 (70.8 - 89.5)	< 0.001
Social relationships	66.7 (50 - 75)	75 (66.6 - 91.6)	< 0.001
Environment	62.5 (53.6 - 71.9)	71.8 (62.5 - 81.2)	< 0.001

Median (1^st^ quartile - 3^rd^ quartile) HF=heart failure; HT=heart transplant

## DISCUSSION

In the present study, it was observed that HT recipients presented higher scores in all domains of the WHOQOL-Bref questionnaire and in the perception of the level of QoL and health. Other studies that also assessed the level of HRQoL between patients with advanced HF and HT recipients also perceived better levels of HRQoL in the last group ^[11-15]^.

Although there was a statistically significant difference between the two groups in all domains of the WHOQOL-Bref questionnaire, an even greater difference is noticeable in the physical domain and in the perception of the level of QoL and health. This fact can be related to the physical limitations imposed by HF in an advanced stage, which limits patients from performing many activities of daily living and, therefore, from having a higher level of HRQoL ^[16]^.

Although the psychological, environmental, and social relationship domains also statistically differed between the two groups, their differences were not as marked as in the other two domains. This may be because these domains were already relatively high in the HF patients group, when compared to the physical domain and the perception of QoL and health. In addition, the signs and symptoms related to HF (such as dyspnea and exercise intolerance) are mostly related to the physical domain, making its most marked difference reasonable.

The impact of heart transplantation in the global QoL can explain the differences seen in domains such as the environmental one - which may erroneously be seen independent of HF symptoms and health status. In fact, the differences observed in covariates such as income and working status suggest that heart transplantation plays a role in many areas of the patients’ lives, which is compatible to the evidenced differences in all domains of HRQoL.

Variables such as sex, age, race, marital status, educational level, and etiology of HF did not significantly differ between the two groups. The study by Czyżewski et al. ^[13]^ (2014), that assessed the level of HRQoL of patients before and after heart transplantation, also identified that variables such as sex and age did not influence the HRQoL of HT recipients.

HT recipients and patients with advanced HF differed in their occupation: the group composed of heart-transplanted patients had a higher percentage of retired individuals and a lower percentage of absence from work due to health problems. In general, after heart transplantation, the rates of return to work are lower, but patients who work after the procedure are less depressed and have fewer general functional, physical, and psychosocial disabilities. Therefore, interventions that assist patients who wish to return to work after heart transplantation should be stimulated ^[17]^.

Although heart transplantation is intended for patients with advanced, refractory HF and with low life expectancy, it should not be seen as a treatment that aims only to survival increase, as it may also offer an improvement in the level of HRQoL ^[18]^.

### Limitations

The cross-sectional design of this study could be considered a limitation. Even though, the analysis showed a significant difference in the level of HRQoL between the groups in pre- and post-transplantation contexts, which contributes to demonstrate the effectiveness of heart transplantation. However, more studies are pertinent to follow prospectively the evolution of the HRQoL of patients with advanced HF who, posteriorly, undergo heart transplantation. An evaluation of the differences between early and late follow-ups is also encouraged.

## CONCLUSION

Patients with advanced HF and HT recipients were compared, according to their level of HRQoL and their social and clinical variables. In the post-transplantation group, it was identified a higher per capita income, a higher percentage of retirees, a lower percentage of absence from work due to health-related issues, and a higher score of HRQoL level in all domains of the WHOQOL-Bref questionnaire, as well as in the perception of the level of QoL and health.

These results make it possible to reaffirm that heart transplantation improves QoL and survival of the recipients and demonstrate an improvement in income after heart transplantation. Although the median age of the HT recipients was that of a middle-aged adult, many patients did not return to work after the transplant and retired. This is probably due to the need for periodic post-transplantation health assessments and to the possibility, guaranteed by Brazilian law, for doing so. Studies that evaluate functional capacity for work in this group should be encouraged, and activities that are appropriate for HT recipients who wish to return to work should be stimulated.

**Table:** 

Authors' roles & responsibilities	
WNC	Substantial contributions to the conception of the work; and the acquisition and analysis of data for the work; drafting the work and revising it; agreement to be accountable for all aspects of the work in ensuring that questions related to the accuracy or integrity of any part of the work are appropriately investigated and resolved; final approval of the version to be published
GSAM	Substantial contributions to the conception of the work; and the acquisition and analysis of data for the work; revising the work; agreement to be accountable for all aspects of the work in ensuring that questions related to the accuracy or integrity of any part of the work are appropriately investigated and resolved; final approval of the version to be published
KCG	Substantial contributions to the conception of the work; and the acquisition and analysis of data for the work; revising the work; agreement to be accountable for all aspects of the work in ensuring that questions related to the accuracy or integrity of any part of the work are appropriately investigated and resolved; final approval of the version to be published
ALM	Substantial contributions to the conception of the work; and the acquisition and analysis of data for the work; revising the work; agreement to be accountable for all aspects of the work in ensuring that questions related to the accuracy or integrity of any part of the work are appropriately investigated and resolved; final approval of the version to be published
MCVM	Substantial contributions to the conception of the work; and the acquisition and analysis of data for the work; drafting the work and revising it; agreement to be accountable for all aspects of the work in ensuring that questions related to the accuracy or integrity of any part of the work are appropriately investigated and resolved; final approval of the version to be published

## References

[1] Bhagra SK, Pettit S, Parameshwar J (2019). Cardiac transplantation indications, eligibility and current outcomes. Heart.

[2] Alraies MC, Eckman P (2014). Adult heart transplant indications and outcomes. J Thorac Dis.

[3] Pelegrino VM, Dantas RAS, Clark AM (2011). Health-related quality of life determinants in outpatients with heart failure. Rev Lat Am Enfermagem.

[4] Wilhelm MJ (2015). Long-term outcome following heart transplantation current perspective. J Thorac Dis.

[5] Fleck MP, Louzada S, Xavier M, Chachamovich E, Vieira G, Santos L (2000). Rev Saude. Publica.

[6] Tackmann E, Dettmer S (2020). Health-related quality of life in adult heart-transplant recipients-a systematic review. Herz.

[7] Alba C, Bain E, Ng N, Stein M, Brien KO, Foroutan F (2016). Int J Transplant Res.

[8] Struck E, Hagl S, Meisner H, Sebening F (1985). Heart transplantation limitations and perspectives. Z Kardiol.

[9] Rosenbaum AN, John R, Liao KK, Adatya S, Colvin-Adams MM, Pritzker M (2014). Survival in elderly patients supported with continuous flow left ventricular assist device as bridge to transplantation or destination therapy. J Card Fail.

[10] Butler J, McCoin NS, Feurer ID, Speroff T, Davis SF, Chomsky DB (2003). Modeling the effects of functional performance and post-transplant comorbidities on health-related quality of life after heart transplantation. J Heart Lung Transplant.

[11] Almenar-Pertejo M, Almenar L, Martínez-Dolz L, Campos J, Galán J, Gironés P (2006). Study on health-related quality of life in patients with advanced heart failure before and after transplantation. Transplant Proc.

[12] Mantovani VM, Silveira CB, Lima LL, Orlandin L, Rabelo-Silva ER, Moraes MA (2017). Comparison of quality of life between patients on the waiting list and heart transplant recipients. Rev Gaucha Enferm.

[13] Czyzewski L, Torba K, Jasinska M, Religa G (2014). Comparative analysis of the quality of life for patients prior to and after heart transplantation. Ann Transplant.

[14] Cannavò A, Passamonti SM, Vincenti D, Aurelio MT, Torelli R, Poli F (2019). Quality of life before and after transplantation in solid organ recipients referred to the North Italy transplant program (NITp) a cross-sectional study. Transplant Proc.

[15] Beilby S, Moss-Morris R, Painter L (2003). Quality of life before and after heart, lung and liver transplantation. N Z Med J.

[16] Heo S, Lennie TA, Okoli C, Moser DK (2009). Quality of life in patients with heart failure ask the patients. Heart Lung.

[17] White-Williams C, Wang E, Rybarczyk B, Grady KL (2011). Factors associated with work status at 5 and 10 years after heart transplantation. Clin Transplant.

[18] Burra P, De Bona M, Germani G, Canova D, Masier A, Tomat S (2007). The concept of quality of life in organ transplantation. Transplant Proc.

